# Helping Opioid Use Disorder and PTSD with Exposure (HOPE): An Open-Label Pilot Study of a Trauma-Focused, Integrated Therapy for OUD/PTSD

**DOI:** 10.3390/bs15070874

**Published:** 2025-06-27

**Authors:** Tanya C. Saraiya, Sonali Singal, Krithika Prakash, Priya Johal, Sara Hameed, Sudie E. Back, Katherine L. Mills, Denise A. Hien

**Affiliations:** 1 Department of Psychiatry and Behavioral Sciences, Medical University of South Carolina, Charleston, SC 29425, USA; backs@musc.edu; 2 Department of Clinical Psychology, Rutgers University-New Brunswick, New Brunswick, NJ 08854, USA; ss3339@gsapp.rutgers.edu (S.S.); sh1934@gsapp.rutgers.edu (S.H.); denise.hien@rutgers.edu (D.A.H.); 3 Department of Psychiatry, University of Pittsburgh, Pittsburgh, PA 15260, USA; prakashk@upmc.edu; 4 Canadian Institute for Substance Use Research, University of Victoria, Victoria, BC V8P 5C2, Canada; pjohal3@student.ubc.ca; 5 The Matilda Centre, University of Sydney, Camperdown, NSW 2050, Australia; katherine.mills@sydney.edu.au; 6 Center of Alcohol & Substance Use Studies, Rutgers University-New Brunswick, New Brunswick, NJ 08854, USA

**Keywords:** opioid use disorder, posttraumatic stress disorder (PTSD), integrated treatment, exposure therapy

## Abstract

Opioid use disorder (OUD) and posttraumatic stress disorder (PTSD) frequently co-occur. However, there are no psychotherapy treatments intentionally designed for this comorbidity, nor designed to be augmented with medications for OUD. In this open-label pilot trial, we tested Helping Opioid Use Disorder and PTSD with Exposure (HOPE), a novel integrated, trauma-focused treatment for individuals (*N* = 6) with OUD/PTSD who were stabilized on medications for OUD. HOPE was delivered weekly for 10–12 sessions, and one follow-up visit was conducted ~1-month post-treatment. Primary outcomes included urine drug screens, the Timeline Followback, Desire for Drugs Questionnaire, Clinician-Administered PTSD Scale-5 (CAPS-5), and PTSD Checklist-5 (PCL-5). Boot-strapped linear mixed effect models and generalized estimating equations showed that PTSD symptoms (CAPS-5: *B* = −7.16, *SE* = 1.24, *p* < 0.01; PCL-5: *B* = −2.04, *SE* = 0.26, *p* < 0.01), desire for opioids (*B* = −0.56, *SE* = 0.15, *p* < 0.01), depression symptoms (*B* = −0.43, *SE* = 0.09, *p* < 0.01), and anxiety symptoms (*B* = −0.50, *SE* = 0.08, *p* < 0.01) decreased significantly over time. Client satisfaction increased throughout the study (*B* = 0.18, *SE* = 0.08, *p* = 0.02), and 83.3% of participants completed the therapy and follow-up visit. There were no significant changes in opioid or other substance use from baseline to follow-up. Although preliminary, results show high acceptability and feasibility of the HOPE therapy and demonstrate significant improvements in PTSD and associated symptoms with an integrated, trauma-focused treatment.

## 1. Introduction

Globally, the opioid epidemic remains a serious public health crisis. In the U.S., from 2002 to 2017, there was a 22-fold increase in the number of deaths involving synthetic opioids (predominantly fentanyl) (Center for Disease Control and Prevention, 2018). A common co-occurring condition with opioid use disorder (OUD) is posttraumatic stress disorder (PTSD), a psychological disorder that can develop following traumatic experiences. Research to date shows that up 50% of treatment-seeking individuals with OUD meet criteria for PTSD (Meier et al., 2014). Medications for OUD (i.e., methadone, buprenorphine, and naltrexone) are considered the gold-standard of treatment for OUD. However, early discontinuation is a major problem (Hser et al., 2014). In addition, medications for OUD do not target co-occurring conditions, and most individuals with OUD/PTSD do not receive treatment for PTSD (Meshberg-Cohen et al., 2019). Indeed, there are no integrated psychotherapy treatments specifically designed for co-occurring OUD/PTSD (Parida et al., 2019; Volkow et al., 2018) despite the prevalence of this comorbidity. To address this gap in clinical care, we developed a new, integrated, trauma-focused psychotherapy for individuals with co-occurring OUD/PTSD and who are stabilized on medications for OUD.

Co-occurring OUD/PTSD shows a distinct comorbidity profile warranting a novel therapy intervention. Research to date shows that, in comparison to other substance use disorders, people with OUD have the highest rates of childhood maltreatment (Sadeh et al., 2021; Santo et al., 2021). An Australian twin study (*N* = 6050) examined twin pairs where one member was exposed to childhood sexual abuse (Nelson et al., 2006). In same-sex twins that reported using illicit drugs, when one member of a twin was exposed to childhood sexual abuse, that twin had an estimated 6-fold increased risk for developing an OUD in adulthood relative to the twin without exposure to childhood sexual abuse. Of note, the risk of developing OUD in adulthood was the highest predictive risk compared to all other substance use disorders (Nelson et al., 2006). This study’s finding suggests that the link between childhood sexual abuse and opioid use is not only stronger than genetic similarities, but also all other possible substance use disorders. Indeed, people with OUD/PTSD show higher rates of overall trauma exposure, including witnessing drug overdoses and death, high rates of chronic pain, and greater cumulative life stressors than those with other co-occurring substance use disorders and PTSD (Becker et al., 2015; Hassan et al., 2017; Santo et al., 2021). People with OUD also have significantly higher rates of PTSD diagnosis and greater PTSD severity relative to those with other primary substance use disorders (Cottler et al., 1992; Mills et al., 2006; Peck et al., 2018). As such, the comorbidity of OUD/PTSD is marked by greater clinical complexity. In comparison to OUD, those with OUD and PTSD have more severe physical health problems, depression, suicidality, pain, unemployment, family concerns, and poorer treatment outcomes, including risk of overdose (Ecker & Hundt, 2018; Hassan et al., 2017; McHugh et al., 2021; Mills et al., 2007; Peck et al., 2021; Schiff et al., 2010).

Recent work has developed theoretical frameworks to explain the frequent co-occurrence of traumatic stress and opioid use. The opioid susceptibility model posits that opioids are sought out to cope with distressing feelings and reactions in response to traumatic experiences and PTSD symptoms (Danovitch, 2016). Studies have supported this theory showing that people with PTSD seek out opioids to manage trauma-related feelings, behaviors, and PTSD symptoms (e.g., Smith et al., 2016). Treatment research also suggests that individuals with OUD/PTSD may particularly benefit from augmenting medications for OUD with psychotherapy. Indeed, people with OUD/PTSD receiving both medications for OUD and psychotherapy addressing OUD had a 4.43 greater odds of abstinence from opioids at end of treatment than those who received medications for OUD only (McHugh et al., 2021). Moreover, failure to treat underlying PTSD in OUD/PTSD has been shown to lead to lower retention on medications for OUD. One study found that for every 10% increase in PTSD symptoms, methadone maintenance attendance decreased by 36% (Peirce et al., 2016).

To date, a handful of studies have shown the benefit of augmenting medications with OUD with trauma-focused treatment. Three pilot studies tested Prolonged Exposure therapy for PTSD (Foa et al., 2019) among individuals with OUD/PTSD and observed significant reductions in PTSD and associated mental health problems (e.g., depression) with no increase in opioid use (Peck et al., 2018; Schacht et al., 2017; Schiff et al., 2015). Most recently, Peck et al. (2025) completed a randomized clinical trial (*N* = 52) examining Prolonged Exposure therapy with contingency management incentives to increase treatment attendance among individuals stabilized on buprenorphine or methadone. Participants with OUD/PTSD and on medications for OUD were randomized to either treatment as usual (i.e., medications for OUD), Prolonged Exposure therapy for PTSD, or Prolonged Exposure with contingency management incentives for attendance. All three groups showed significant reductions in PTSD symptoms with no group differences, but the contingency management group showed the greatest improvement in PTSD symptoms among all three groups and the highest level of treatment attendance. Moreover, both Prolonged Exposure therapy groups showed no increase in substance use (Peck et al., 2025). Taken together, the findings demonstrate promise for this modified Prolonged Exposure therapy among people with OUD/PTSD. However, a limitation of the studies to date is that none have integrated psychotherapy content on both OUD and PTSD in one treatment manual (i.e., other manuals have solely focused on one condition, namely PTSD). A single treatment incorporating content on both OUD and PTSD may be important given the reciprocal feedback loop between posttraumatic stress and substance use (Haller & Chassin, 2014; Khoury et al., 2010; Nelson et al., 2006).

The current study tested the feasibility and preliminary efficacy of a new manualized psychotherapy, Helping Opioid Use Disorder and PTSD with Exposure (HOPE). Informed by the NIDA Phase Model for Intervention Development (Onken et al., 2014), HOPE was developed following mixed methods feedback from community stakeholders and patients with lived experience (Saraiya et al., 2024). HOPE was also informed by prior research on integrated treatments targeting other substance use disorders and PTSD (Back et al., 2014). Of the studies examining interventions for OUD/PTSD, all have focused primarily on PTSD with limited to no psychosocial intervention content targeting OUD, the co-occurrence of OUD/PTSD, or medication for OUD (Peck et al., 2025; Peck et al., 2018; Schacht et al., 2017; Schiff et al., 2015). HOPE was designed to include content addressing (a) PTSD symptoms, (b) the co-occurrence of OUD/PTSD, (c) opioid use, other substance use, and craving; and (d) adherence to medications for OUD. In this open-label pilot trial, HOPE was evaluated among individuals with co-occurring OUD/PTSD who were stabilized on medications for OUD. The aims of the study were to test if participants receiving HOPE reported (1) significant reductions in their PTSD symptoms; (2) significant reductions in their opioid use and craving; (3) retention on medications for OUD; and (4) client satisfaction with the treatment. Secondary analyses examined if participants receiving HOPE reported significantly reduced symptoms of anxiety and depression.

## 2. Methods

### 2.1. Participants

Participants (*N* = 6) were treatment-seeking adults with co-occurring OUD/PTSD. They were referred to the study by providers from local substance use treatment clinics and recruited from social media advertisements between October 2023 and April 2024. To be included, participants had to (1) be at least 18 years of age; (2) English speaking; (3) meet Diagnostic and Statistical Manual of Mental Disorders, Fifth Edition (DSM-5; American Psychiatric Association, 2013) criteria for current (i.e., past year) OUD or lifetime OUD in the past 5 years; (4) meet DSM-5 criteria for current PTSD (American Psychiatric Association, 2013); and (5) be maintained on a stable dose of medication for OUD and, if applicable, any psychotropic medications for at least 1 month prior to study procedures. Exclusion criteria included (1) unmanaged (i.e., not controlled with medications and/or experiencing active symptoms) psychosis, mania, or bipolar disorder; (2) past year suicidal ideation with a plan and intent and/or past year suicide attempt; (3) meeting DSM-5 diagnostic criteria for a primary current non-opioid substance use disorder; (4) being enrolled in ongoing evidence-based psychotherapy for substance use disorders or PTSD outside of standard psychotherapy groups in substance use treatment settings, and (5) having medical problems requiring immediate and intensive treatment. All study procedures were approved by the affiliated institution’s Institutional Review Board.

### 2.2. Procedures

**Figure 1:**
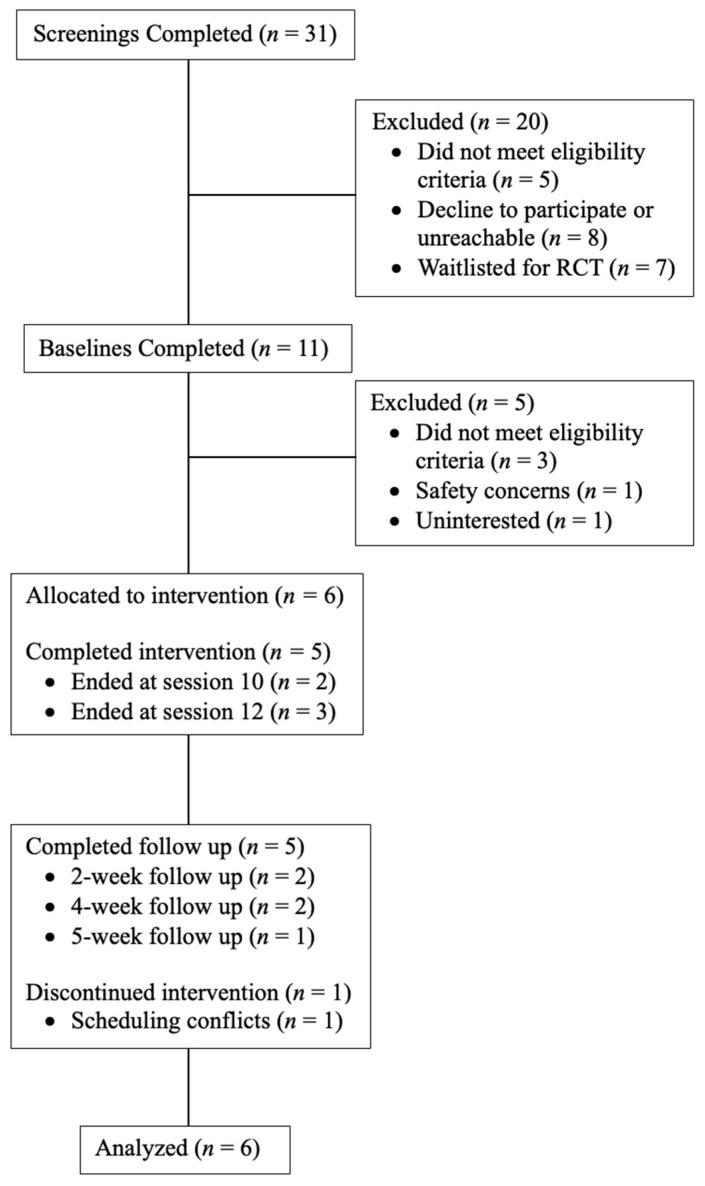
Consort Flow diagram.

Interested individuals completed an initial phone screening, provided written informed consent, and completed a baseline appointment to determine study eligibility. Eligible individuals received 10–12 individual 60 min sessions of HOPE with a licensed clinical psychologist (first author) once or twice per week, depending on the participant’s schedule and desired treatment frequency. Trained clinical psychology doctoral students conducted weekly self-report assessments in addition to clinical interviews at baseline, week 6, end of treatment (i.e., a variable time point that aligned with either session 10 or 12 for each participant), and ~1 month after end of treatment (i.e., follow up; see below for more information). Treatment completers (*n* = 5; 83.3%) were defined as completing at least 10 psychotherapy sessions. The one participant who did not complete the study dropped out at session 4. Multiple attempts were made to reschedule the participant, but the participant was unreachable. As such, end of treatment and follow up assessments were not completed with this one participant. Participants were compensated for their time and completion of assessments at a rate of USD 50 for baseline, USD 25 for weekly therapy sessions, and USD 50 for 1-month follow up. Figure 1 illustrates the study design and participant retention.

### 2.3. The HOPE Intervention

Helping Opioid Use and PTSD with Exposure (HOPE) includes modified components of exposure therapies to address trauma/PTSD (Foa et al., 2019; Sloan & Marx, 2019) and relapse prevention therapy (Kadden et al., 1992) to address substance use disorders, including OUD. HOPE is delivered in an individual format for 60 min a week; it comprises 10 to 12 sessions. To determine treatment length, patients and providers collaboratively discussed progress in therapy at session 8–9 and then jointly decided on overall treatment length. Major modifications from the parent intervention (COPE; Back et al., 2014) to HOPE include (a) increased flexibility in treatment sessions such that the therapist may choose what to focus on in session; (b) flexibility in treatment length (i.e., 10 to 12 sessions), (c) shorter sessions (i.e., 60 min vs. 90 min); (d) integration of content on opioid use/OUD, medication for OUD, opioid-related overdose, chronic pain, and the relationship between traumatic stress and opioid use; (e) expansion of exposure therapy to include multiple traumas; (f) a reduced number of imaginal and in vivo exposures; (g) reduction in homework; (h) integration of content on general life stressors; and (i) OUD safety and risk planning. In HOPE, imaginal exposures begin at session 4, but imaginal and in vivo exposures are not performed in every session following session 4. Rather, both are titrated. For imaginal exposure, at least three exposures over three sessions were completed over the course of treatment with each exposure lasting a minimum of 15 min. After three imaginal exposures, subsequent imaginal exposures could also include other traumatic events. Similarly, in vivo exposures were titrated based on participant ability to complete such an activity. Further, in vivo exposures were not required to be 30–45 min in length or repeated multiple times between sessions.

In regard to structure, HOPE mirrors the Trauma-Focused Cognitive Behavioral Therapy (TF-CBT; Cohen & Mannarino, 2015) model in that there are four domains to address throughout the therapy: Psychoeducation, Relapse Prevention, Exposure, and Processing (PREP). The therapy intervention includes suggested organization of content within each major domain (i.e., PREP), but therapists have flexibility in choosing which domains to focus on in each session. Overall benchmarks are provided to balance treatment fidelity with flexibility (i.e., complete at least 3 exposures on the index traumatic event before moving to subsequent traumas) and thus aim greater implementation and personalized treatment success (Chu & Kendall, 2009; Galovski et al., 2024). Importantly, sessions are meant to respond to patient presentation and needs while also ensuring safety. For this open-label pilot study, the first author was the study therapist, and fidelity checklists were completed after each session.

### 2.4. Measures

**Demographics**. Participant demographics were assessed at baseline using a study-developed questionnaire that gathered information on age, gender identity, racial/ethni identity, religious affiliation, education level, monthly household income, legal history, housing and relationship status, and childcare responsibilities.

**Clinical Diagnoses.** At baseline, trained assessors completed the Structured Clinical Interview for DSM-5 Disorders (SCID; First et al., 2015) for substance use disorders, mood disorders (i.e., depression, mania, bipolar), and psychosis.

**Trauma Exposure**. Trauma exposure was assessed by the Life Events Checklist-5 (LEC-5; Weathers et al., 2013a). The LEC-5 is a self-report measure designed to assess an individual’s exposure to 16 different types of potentially traumatic events. These events are broadly categorized into natural disasters, accidents, interpersonal violence, and other traumatic experiences, such as the death of a loved one or witnessing violence. The measure is used as part of the PTSD diagnostic process to identify trauma exposure, which may then be linked to the presence of trauma-related symptoms. Respondents indicate varying levels of exposure to each type of potentially traumatic event (i.e., happened to me, witnessed it, learned about it, part of my job, not sure, doesn’t apply), and respondents may endorse multiple levels of exposure to the same trauma type. Participants were asked which of the endorsed events was their worst (i.e., index) trauma for subsequent assessments.

Childhood trauma was also assessed by the childhood trauma questionnaire (CTQ; Bernstein & Fink, 1998). The CTQ is a self-report measure designed to assess childhood abuse and neglect across five domains: emotional abuse, physical abuse, sexual abuse, emotional neglect, and physical neglect. The CTQ consists of 28 items, rated on a 5-point Likert scale (1 = Never true, 5 = Very often true), assessing the severity of each type of trauma. It includes 5 subscales: Emotional Abuse, Physical Abuse, Sexual Abuse, Emotional Neglect, and Physical Neglect. Each item is scored from 1 to 5, and the subscales are totaled to provide an overall score for each category of trauma. Each subscale can range from 5 to 25 where higher scores reflect greater severity of trauma in that domain. The total scores are then compared against established cutoff values to categorize the severity of trauma (e.g., None, Low, Moderate, Severe; Bernstein & Fink, 1998).

**PTSD Severity**. PTSD symptoms were assessed by the Clinician-Administered PTSD Scale for DSM-5 (CAPS-5; Weathers et al., 2018) at baseline, mid-treatment, end-of-treatment, and follow-up and the PTSD Checklist-5 (PCL-5; Blevins et al., 2015; Weathers et al., 2013b) at every time point. The CAPS-5 is a 30-item structured diagnostic interview for assessing PTSD diagnostic status and severity. It is regarded as the gold standard for PTSD assessment due to its evaluation of both symptom frequency and intensity corresponding to the DSM-5 diagnostic criteria for PTSD. These symptoms are queried in response to a criterion A traumatic event and cover criterion B (intrusion; 5 items), criterion C (avoidance; 2 items), criterion D (negative cognitions and emotions; 7 items), criterion E (arousal and reactivity; 6 items), and dissociative subtype (depersonalization and derealization; 2 items) symptoms. Trained doctoral-level students rated each item on a 5-point scale ranging from 0 (none) to 4 (extreme) and combined information about frequency and intensity of each item into a single severity rating. A PTSD diagnosis requires meeting the DSM-5 criteria of at least one criterion B symptom, one criterion C symptom, two criterion D symptoms, and two criterion E symptoms, all with severity scores ≥2. A total symptom severity score was calculated by summing the scores for the 20 DSM-5 PTSD symptoms, and a dissociative subtype can be determined when scores are ≥2 for either the depersonalization or derealization item. Total scores can range from 0 to 80 with higher scores denoting greater symptom severity.

The PTSD Checklist for DSM-5 (PCL-5) is a 20-item self-report measure that assesses the presence and severity of PTSD symptoms according to DSM-5 criteria (Blevins et al., 2015; Weathers et al., 2013b). Participants rated the degree to which they have been bothered by each symptom in response to their criterion A traumatic event over the assessment period using a 5-point Likert scale ranging from 0 (not at all) to 4 (extremely). Scores from all items are summed to yield a total severity score ranging from 0 to 80, with higher scores indicating greater symptom severity. A cut-off score of ≥33 is used to indicate a provisional PTSD diagnosis and criterion severity scores can be calculated by summing the items within each respective symptom cluster. In this study, the PCL-5 was administered at baseline to determine past-month PTSD symptom severity, at each weekly session throughout the 12-week treatment period, and at the 1-month post-treatment follow-up.

**Substance Use and Medication for OUD**. Substance use was measured by self-report and biochemical verification through the Timeline Followback (TLFB; Sobell & Sobell, 2000) TLFB and a saliva-based alcohol test strip and an 11-panel urine drug screen (UDS) at each time point (e.g., baseline, weekly assessments, end of treatment, and follow-up). An Alco-Screen saliva test strip was used at each assessment period for the detection of blood alcohol. In addition, an 11-panel urine drug screen detected the presence of drugs or their metabolites in participant urine samples. Urine drug screens tested for the following: amphetamines (AMP), barbiturates (BAR), buprenorphine (BUP), benzodiazepines (BZO), cocaine (COC), methamphetamine (MET), methadone (MTD), opiates (OPI/MOR 300), oxycodone (OXY), cannabis (THC), and fentanyl (FEN). Of note, fentanyl test strips were in separate urine drug screen dip cards. The UDS provided binary (positive/negative) results for each drug. If a participant tested positive for any drug, it was also assessed with the TLFB.

The TLFB is a retrospective calendar assessment tool used to collect detailed information about an individual’s substance use over a specified period (e.g., the past month, 3 months, etc.). The calendar method helps individuals recall the frequency, quantity, and context of their substance use by providing a timeline to aid memory recall. At baseline, study participants completed a past 60-day TLFB with a study team member. At each weekly visit, the TLFB was used to collect information about the individual’s substance use since the last visit. At follow-up, the TLFB was used to assess substance use since the last treatment session. Substance use was quantified as average days of use in the past assessment period for overall opioid misuse and all other substance use (e.g., alcohol, tobacco, stimulants, etc.).

Following NIH recommendations, the study protocol employed a dual verification approach combining patient self-report with biospecimen analysis via urine drug screening to confirm or disconfirm self-reported medication for OUD (Biondi et al., 2020). Medication for OUD adherence was assessed using a study-developed measure that documented medication type, prescribed dosage, and adherence status. At each assessment point, the assessors recorded whether the prescribed dose was taken during the preceding assessment period. When doses were missed—which only occurred to two participants in the study—specific reasons for non-adherence were systematically queried. In addition, both the TLFB and UDS were also used to verify adherence to medication for OUD. Positive UDS for the participant’s self-reported medication for OUD was compared with the TLFB and verified with the participant for adherence to medication for OUD or potential misuse. In only one participant, at one assessment period, was there a discrepancy between the participant self-report and UDS for medication for OUD. In cases where participants discontinued medication for OUD entirely, study staff had release of information to consult with the prescribing provider. Further, non-fatal overdoses were assessed at each assessment time point; there were no non-fatal overdoses during the study.

**Opioid Craving.** Opioid craving was measured by the Desire for Drug Questionnaire (DDQ; Franken et al., 2002) and the Opioid Craving Scale (OCS; McHugh et al., 2014). The DDQ is a 14-item measure originally designed to assess for three factors of heroin use: desire and intention, negative reinforcement, and control. It was modified for this study to focus on all opioid use. Questions (e.g., “I would accept to use opioids now if it was offered to me”) assess for desire for opioid use on a 7-point Likert scale ranging from 1 = Strongly disagree to 7 = Strongly agree. Psychometric research shows strong reliability and validity on patients with substance use disorders (Franken et al., 2002). The DDQ was administered at baseline, mid-treatment, end of treatment, and follow-up. Solely, the desire for opioids subscale was used for study analyses. The OCS is a brief, 3-item measure for opioid craving. Responses range from 1 to 10 on a Likert scale (1 = not at all, 10 = extremely) and assess for current craving, past week craving, and environmental craving. The OCS was administered at every assessment time point.

**Client Satisfaction**. The Client Satisfaction Questionnaire (CSQ-8) is an 8-item, unidimensional self-report measure designed to assess general satisfaction with and perceived effectiveness of health services (Attkisson & Zwick, 1982). The measure evaluates various aspects of service satisfaction including quality of service, meeting client needs, recommendation to others, and willingness to return for services. Each item is rated on a 4-point Likert scale and scores are summed to produce a total score ranging from 8 to 32, with higher scores indicating greater satisfaction with services. The CSQ-8 has demonstrated excellent psychometric properties across diverse clinical populations, including strong internal consistency (Cronbach’s α = 0.93), good test–retest reliability, and strong correlations with changes in client-reported symptoms (Attkisson & Zwick, 1982; Nguyen et al., 1983). The CSQ-8 was administered at the end of each weekly therapy session to identify changes in client satisfaction over the course of treatment and to inform refinement of the treatment for further evaluation.

**Depression and Anxiety Symptoms**. Two measures were used to assess symptoms of anxiety and depression. The Generalized Anxiety Disorder 7 (GAD-7; Spitzer et al., 2006) is a 7-item self-report questionnaire used to screen for generalized anxiety disorder. It assesses the frequency of symptoms of anxiety over the last two weeks. Each of the 7 items is rated on a 4-point Likert scale (0 = Not at all, 1 = Several days, 2 = More than half the days, 3 = Nearly every day), assessing the severity of anxiety symptoms (e.g., feeling nervous, worrying excessively, or becoming easily upset). The scores for each item are summed to produce a total score (0–21): 0–4: Minimal anxiety; 5–9: Mild anxiety; 10–14: Moderate anxiety; 15–21: Severe anxiety. In addition, the Patient Health Questionnaire-9 (PHQ-9; Kroenke & Spitzer, 2002) is a 9-item self-report tool used to screen for depression and assess the severity of depressive symptoms over the past two weeks. Each item is rated on a 4-point Likert scale (0 = Not at all, 1 = Several days, 2 = More than half the days, 3 = Nearly every day), based on the frequency of symptoms of depression (e.g., little interest or pleasure in doing things, feeling down or hopeless). The scores for each item are summed, with total scores ranging from 0 to 27. Severity is ranked as 0–4: minimal depression; 5–9: mild depression; 10–14: moderate depression; 15–19: moderately severe depression; 20–27: severe depression.

### 2.5. Data Analysis

Data analyses examined trajectories for the following outcome variables over the course of treatment—PTSD symptoms (PCL-5 and CAPS-5), opioid and other substance use (UDS and TLFB), opioid craving (DDQ), client satisfaction (CSQ-8), medication for OUD retention (TLFB), depression (PHQ-9), and anxiety (GAD-7). All variables were measured at baseline (pre-treatment), each treatment session, end of treatment, and one-month follow-up, except for the CAPS-5 and DDQ, which were assessed at baseline, mid-treatment (week 6), end of treatment, and at follow-up. All analyses examined the intent-to-treat sample (*N* = 6) except for the CAPS-5 and DDQ, due to the one participant who dropped out solely having baseline data on these two measures. The TLFB assessed use of each substance separately, tracking the number of times, amount, and route of administration each substance was used since the last assessment time point. The timeframe for the last assessment point varied—for instance, at baseline, it covered the past 60 days, while at the one-month follow-up, it covered the past 30 days. In some cases, participants had a two-week gap between sessions, resulting in a 14-day assessment period instead of the usual 7 days. Therefore, to standardize this variable, during processing, use was divided by the number of days since the last assessment, to calculate average using days.

Data analysis was conducted in R version 4.4.2 using the following packages for data manipulation, visualization, and statistical modeling: haven, tidyverse, lme4, emmeans and geepack (Bates et al., 2015; Højsgaard et al., 2024; Lenth, 2025; Wickham et al., 2019; Wickham et al., 2023). To assess outcomes over time, we utilized different modeling approaches based on the nature of the variables. Given that all variables, except for the UDS results, were largely continuous, we utilized a linear mixed-effects model (LME) and included random intercepts for participants to account for repeated measures of time. For each model, time was first treated as a continuous variable to assess overall trends and then as a categorical variable to calculate estimated marginal means (EMMs) for each time point, to achieve a more nuanced understanding of treatment trajectories over time. When significant effects were found, all estimates were then bootstrapped to obtain bias-corrected confidence intervals and robust p-values to ensure reliability of results, given the small sample size. Adjusted within-group effect sizes in the form of Hedges g were calculated with confidence intervals.

Due to the categorical nature of UDS (binary for opioid use and count-based for non-opioid use), we implemented generalized estimating equations (GEE) to assess population-level trends. Given the low number of positive UDS results for both opioids and all other substance use, identifying individual trajectories was challenging, so the focus remained on estimating overall population-level effects rather than individual variation, which also makes GEE a better fit (Ballinger, 2004). For opioid use, which had a binary outcome, a logistic regression model was fitted using GEE. For non-opioid use, which was a count variable, model selection between Poisson and negative binomial regression depended on the presence of overdispersion, which was assessed by comparing the mean to the variance (Gardner et al., 1995). Lastly, key model parameters were summarized for clarity, and graphs were created to visualize significant results.

## 3. Results

### 3.1. Demographics

Table 1 and Table 2 shows participant demographics and clinical characteristics, respectively. The sample identified predominantly as female (66.7%), white (66.7%), on Medicaid (83.3%), with an incarceration history (66.7%), and as parents (66.7%). The most common medication for OUD was buprenorphine (66.7%) followed by methadone (16.7%) and vivitrol (16.7%). On average, participants experienced and witnessed *M* = 8.67 (SD = 1.45) lifetime traumatic events; index traumas included sexual assault (50.0%), physical assault (33.3%), and sudden violent death (16.7%). A majority of the sample reported using nicotine products (83.3%) with fewer participants reporting ongoing cocaine use (16.6%; *n* = 1) and methamphetamine use (16.6%; *n* = 1).

### 3.2. PTSD Outcomes

**Figure 2:**
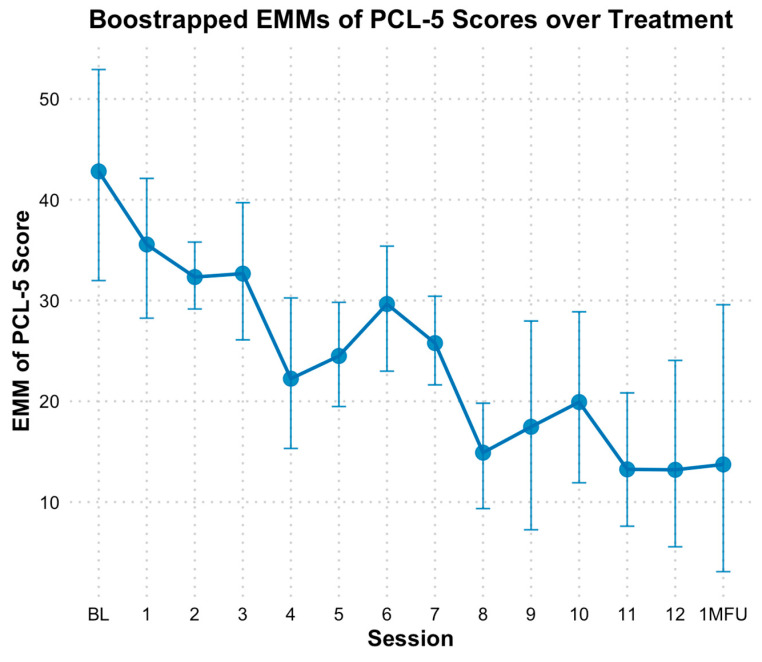
Bootstrap estimated marginal means of PCL-5 scores over treatment. Note: Error bars represent 95% bootstrap confidence intervals. PCL-5 = Posttraumatic Stress Disorder Checklist for DSM-5; EMM = estimated marginal means. The x-axis reflects sessions (primarily weekly) of treatment, with 0 = baseline, 1 = session 1, etc. 1MFU = is the 1 month follow up assessment which ranged from 2-5 weeks after end of treatment.

Based on the PCL-5, results indicated a significant reduction in PTSD symptoms over time, with bootstrapped estimates showing that PCL-5 total scores decreased by approximately 2.06 points per session (*B* = −2.06, *SE* = 0.26, *p* < 0.01). The fixed effect of time alone explained 24% of the variance in PCL-5 scores, and the full model, including individual baseline differences, explained 75% of the variance. When time was analyzed as a categorical variable, bootstrapped estimated marginal means (EMMs) for PCL-5 scores were lower at nearly all sessions following baseline (*M* = 42.33, *SE* = 5.96). However, significant differences based on bootstrapped confidence intervals (CIs) were observed at sessions 4, 5, and 7 through 13 compared to baseline, with the most pronounced decline occurring at session 8 (see Figure 2). Specifically, PCL-5 scores at mid-point (session 6) did not indicate significant decline (*M* = 29.72, *SE* = 0.06, *Hedges g* = 1.21 [0.24, 2.36]) based on bootstrapped CIs; however, end of treatment (*M* = 13.35, *SE* = 0.15, *Hedges g* = 2.78 [1.08, 4.99]), and one-month follow-up (*M* = 13.71, *SE* = 0.02, *Hedges g* = 2.74 [1.07, 4.93]) reflected significant declines from baseline.

**Figure 3:**
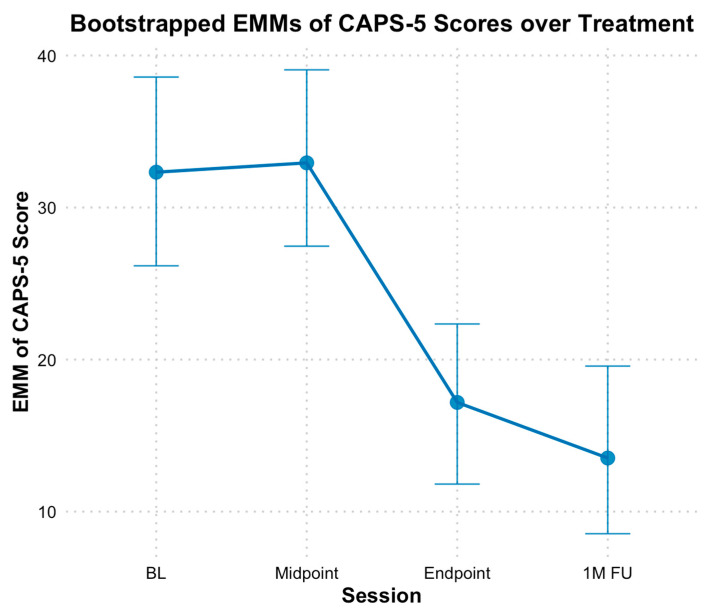
Bootstrap estimated marginal means of CAPS-5 scores over treatment. Note: Error bars represent 95% bootstrap confidence intervals. CAPS-5 = Clinician Administered Posttraumatic Stress Disorder Scale for DSM-5; EMM = estimated marginal means; BL = Baseline; 1MFU = 1-month follow-up.

With the CAPS-5, the analysis revealed a significant decrease in PTSD symptoms from baseline through the mid-point, endpoint, and follow-up, with bootstrapped estimates indicating an average reduction of 7.16 points between these time points (*B* = −7.16, *SE* = 1.24, *p* < 0.01). The fixed effect of time alone explained 37% of the variance in CAPS scores, and the full model, including individual baseline differences, explained 79% of the variance. Additionally, when time was treated as a categorical variable, no significant difference based on bootstrapped CIs was found between EMMs of CAPS-5 scores at baseline (*M* = 32.20, *SE* = 2.96) and mid-point (*M* = 32.80, *SE* = 2.78, *Hedges g* = −0.10 [−0.80, 0.60]) scores; however, both endpoint (*M* = 17.00, *SE* = 2.63, *Hedges g* = 2.40 [0.89, 4.34]) and follow-up EMM CAPS-5 scores (*M* = 13.60, *SE* = 2.78, *Hedges g* = 2.94 [1.16, 5.26]) were significantly lower compared to baseline (see Figure 3).

### 3.3. Opioid and Other Substance Use Outcomes

Based on the TFLB, opioid use results indicated no significant change in outcomes over time. When time was treated as a continuous variable, the fixed effects for TLFB opioid use per day since the last time point (*B* = −0.0017, *SE* = 0.002, *p* = 0.40) was not statistically significant. The fixed effect of time alone explained 0.99% of the variance in TLFB opioid use per day, and the full model, including individual baseline differences, explained 2.8% of the variance. Similarly, when time was modeled as a categorical variable, no consistent significant differences from baseline emerged across sessions. Although average opioid use per day since the last assessed time point appeared elevated at session 8 (*M* = 0.14 days, *SE* = 0.03, *Hedges g* = −1.76 [−3.26, −0.56]) compared to baseline (*M* = 0.006, *SE* = 0.03), this was largely attributable to opioid use in two patients, and bootstrapped CIs also indicate that this difference was not statistically significant. TLFB opioid use per day at mid-point (session 6; *M* = −0.000031, *SE* = 0.01, *Hedges g* = 0.07 [−0.63, 0.78]), end of treatment (*M* = 0.0006, *SE* = 0.01, *Hedges g* = 0.06 [−0.64, 0.77]) and one-month follow-up (*M* = −0.00003, *SE* = 0.01, *Hedges g* = 0.07 [−0.63, 0.78]) did not reflect statistically significant changes from baseline either.

When time was treated as a continuous variable, the fixed effects for TLFB non-opioid substance use per day since the last time point (*B* = −0.0006, *SE* = 0.006, *p* = 0.80) was not statistically significant. For TLFB non-opioid substance use per day, the fixed effect of time alone explained 0.02% of the variance; however, the full model, which included individual baseline differences, explained 78.5% of the variance. Similarly, when time was modeled as a categorical variable, no consistent significant differences from baseline emerged across sessions. Average non-opioid substance use per day since the last assessed time point was lowest at session 7 (*M* = 0.52, *SE* = 0.03, *Hedges g* = 1.40 [0.35, 2.67]) compared to baseline (*M* = 0.84, *SE* = 0.18). TFLB non-opioid substance use per day scores at mid-point (session 6; *M* = 0.67, *SE* = 0.18, *Hedges g* = 0.74 [−0.07, 1.63]), end of treatment (*M* = 0.86, *SE* = 0.20, *Hedges g* = −0.08 [−0.78, 0.62]) and one-month follow-up (*M* = 0.84, *SE* = 0.18, *Hedges g* = 0.006 [−0.69, 0.71]) did not reflect significant changes from baseline. However, bootstrapping failed to converge, likely due to insufficient variability in the data, indicating that these estimates may be unstable or unreliable.

Regarding UDS data, results also indicated no significant change in opioid and non-opioid use over time. When time was modeled continuously, the log-odds of average opioid use per day over time did not significantly change (*B* = −0.012, *SE* = 0.01, *p* = 0.34). Given that there was no evidence for overdispersion (*M* = 0.46, *Var* = 0.43), a Poisson model was used for non-opioid substance counts, but the time effect was not significant (*B* = −0.02, *SE* = 0.01, *p* = 0.12). When time was modeled categorically, no meaningful results emerged for opioid use, likely due to the high presence of zero values (no presence of opioids in urine screen) across sessions. Additionally, the non-opioid substance UDS model failed to converge when time was modeled categorically.

### 3.4. Opioid Craving and Desire

Based on the OCS measure, results indicated slight reduction in opioid craving over time during treatment. When modeling time as a continuous variable, bootstrapped estimates showed the change was non-statistically significant (*B* = −0.098, *SE* = 0.057, *p* = 0.08). The fixed effect of time alone explained 2.52% of the variance in the OCS scores; however, the full model, which included the individual baseline differences, explained 44.95% of the variance. When time was analyzed categorically, EMMs for OCS scores suggested variability across sessions, with craving scores lower at later sessions compared to baseline. There was no statistically significant difference in scores at midpoint (session 6; *M* = 2.37, *SE* = 0.72, *Hedges g* = 0.60 [−0.17, 1.44]) or end of treatment (*M* = 1.10, *SE* = 0.02, *Hedges g* = 1.16 [0.21, 2.28]) compared to baseline (*M* = 3.73, *SE* = 1.04), based on bootstrapped CIs. However, statistically significant differences in EMMs emerged at the one-month follow up (*M* = 0.57, *SE* = 1.04, *Hedges g* = 1.39 [0.35, 2.66]) compared to baseline.

**Figure 4:**
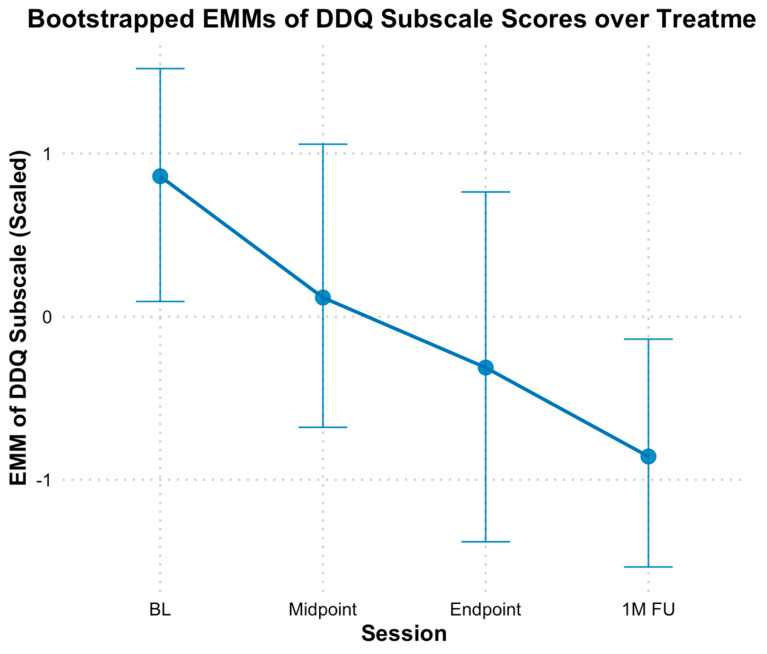
Bootstrap estimated marginal means of Desire and Intention for opioid use subscale scores from DDQ over treatment. Note: Error bars represent 95% bootstrap confidence intervals. DDQ—Drug Desire Questionnaire; EMM = estimated marginal means; BL = Baseline; 1MFU = 1-month follow-up.

Based on the Desire and Intention subscale of DDQ, results revealed a significant decline in participants’ desire and intention to use opioids over time (Figure 4). When modeling time continuously, bootstrapped estimates indicated a significant decrease in scores by approximately 0.56 standardized points per assessment (*B* = −0.56, *SE* = 0.15, *p* < 0.01). The fixed effect of time alone explained 39.54% of the variance in the Desire and Intention subscale, and the full model, including individual baseline differences, explained 42.67% of the variance. Additionally, when analyzed categorically, bootstrapped EMMs generally decreased at each subsequent assessment point from baseline (*M* = 0.86, *SE* = 0.35), there were no significant differences at mid-point (*M* = 0.11, *SE* = 0.45, *Hedges g* = 0.74 [−0.07, 1.63]), and end of treatment (*M* = −0.32, *SE* = 0.52, *Hedges g* = 1.15 [0.20, 2.27]) compared to baseline, based on bootstrapped CIs. However, significant differences were observed at the one-month follow-up (*M* = −0.85, *SE* = 0.37, *Hedges g* = 1.66 [0.50, 3.10]) compared to baseline. 

### 3.5. Medication for OUD Retention

Based on TFLB, there was no evidence of significant change in medication for OUD retention over time, suggesting that participants did not show significant decreases in retention on medication for OUD from baseline. When modeling time as a continuous variable, the fixed effect for average daily medications for OUD use since the previous time point was not statistically significant (*B* = −0.006, *SE* = 0.006, *p* = 0.30). The fixed effect of time alone explained 1.11% of the variance in TLFB medication for OUD, and the full model, including individual baseline differences, explained 33.13% of the variance. Similarly, no significant differences emerged when modeling time categorically across sessions. TFLB for average daily medications for OUD use at mid-point (session 6; *M* = 1.00, *SE* = 0.10, *Hedges g* = −0.39 [−1.16, 0.34]), end of treatment (*M* = 1.05, *SE* = 0.12, *Hedges g* = −0.58 [−1.42, 0.18]), and one-month follow-up (*M* = 0.91, *SE* = 0.10, *Hedges g* = 0.03 [−0.68, 0.73]) did not show statistically significant differences from baseline. Bootstrapping was not conducted due to the lack of significant findings.

### 3.6. Client Satisfaction

**Figure 5:**
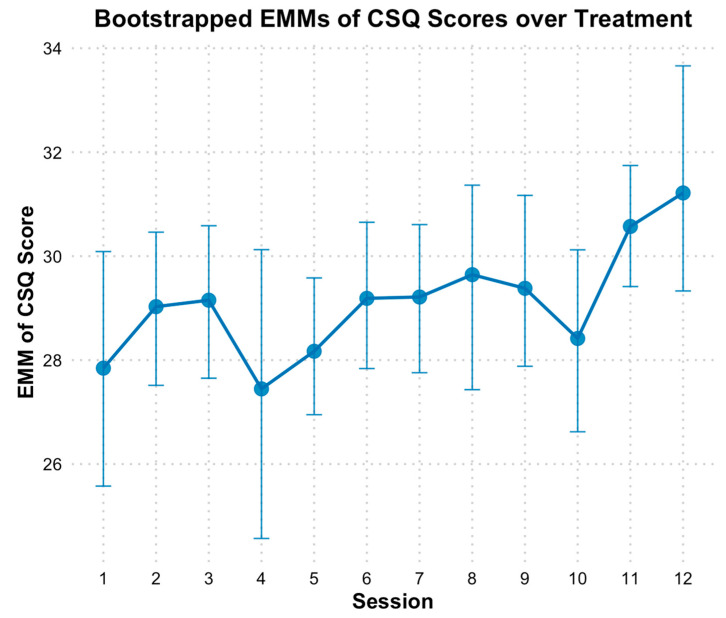
Bootstrap Estimated Marginal Means of CSQ Scores Over Treatment. Note: Error bars represent 95% bootstrap confidence intervals. CSQ = Client Satisfaction Questionnaire; EMM = estimated marginal means. The x-axis reflects sessions of treatment, with 0 = baseline, 1 = session 1, etc.

Based on the CSQ-8, there was evidence of a significant linear increase in client satisfaction over treatment (Figure 5). Modeling time as a continuous predictor revealed bootstrapped estimates indicating that CSQ scores increased by approximately 0.18 points per session (*B* = 0.18, *SE* = 0.08, *p* = 0.02). The fixed effect of time alone explained 4.95% of the variance in CSQ scores, and the full model, including individual baseline differences, explained 54.92% of the variance. When time was analyzed categorically, EMMs of client satisfaction scores generally showed increasing satisfaction across sessions compared to baseline, except for session 4, which coincided with the initiation of initially distressing imaginal exposure sessions. However, bootstrapped CIs on the EMM scores indicated no significant differences across time points. It is important to note that the CSQ typically ranges from 8 to 32, and satisfaction was already high at session 1 (*M* = 27.83, *SE* = 1.11). This mean value suggests that participants were “mostly” to “very satisfied” across key areas of HOPE, such as the quality of the therapy, how well the therapy met their needs, and their willingness to recommend the therapy to others. Consequently, despite the absence of statistically significant differences between sessions, satisfaction scores approached the maximum score by session 12 (*M* = 31.24, *SE* = 1.38, *d =* 1.81; Figure 5).

### 3.7. Depression and Anxiety

**Figure 6:**
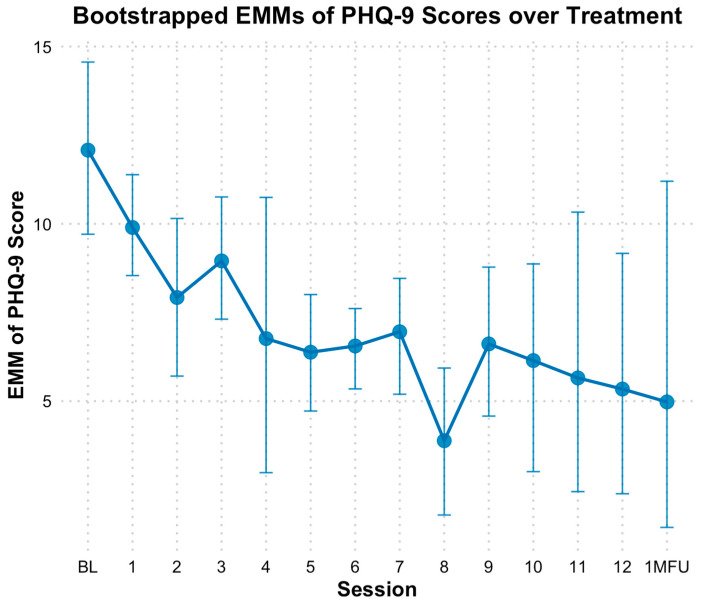
Bootstrap estimated marginal means of PHQ-9 over treatment. Note: Error bars represent 95% bootstrap confidence intervals. PHQ-9 = Patient Health Questionnaire; EMM = estimated marginal means. The x-axis reflects sessions (primarily weekly) of treatment, with 0 = baseline, 1 = session 1, etc. 1MFU = is the 1 month follow up assessment which ranged from 2 to 5 weeks after end of treatment.

Based on the PHQ-9, there was a significant reduction in depressive symptoms over time. When modeling time as a continuous variable, bootstrapped estimates indicated a significant decrease in PHQ-9 scores by approximately 0.43 points per session (*B* = −0.43, *SE* = 0.09, *p* < 0.01). The fixed effect of time alone explained 9.77% of the variance in PHQ-9 scores; However, the full model which included individual baseline differences, explained 73.83% of the variance. When analyzed categorically, bootstrapped EMMs revealed consistent declines in PHQ-9 scores across sessions, with significant differences based on bootstrapped CIs observed at sessions 5 through 10, session 12, and follow-up compared to baseline (Figure 6). Specifically, PHQ-9 scores at mid-point (session 6; *M* = 6.57, *SE* = 0.56, *Hedges g* = 1.53 [0.43, 2.88]), end of treatment (*M* = 5.37, *SE* = 1.76, *Hedges g* = 1.87 [0.62, 3.45]) and one-month follow-up (*M* = 4.97, *SE* = 2.72, *Hedges g* = 1.98 [0.68, 3.64]) reflected significant reductions from baseline (*M* = 12.00, *SE* = 1.24).

**Figure 7:**
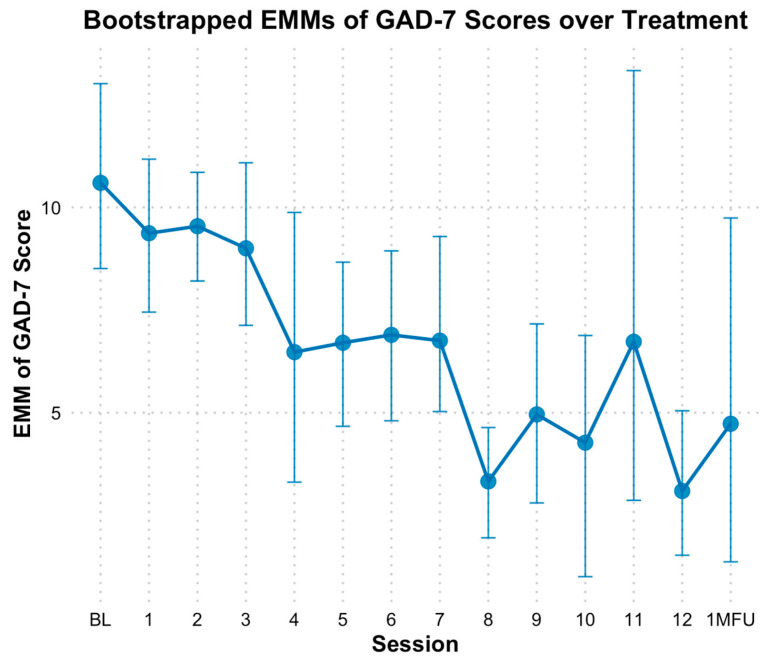
Bootstrap estimated marginal means of GAD-7 over treatment. Note: Error bars represent 95% bootstrap confidence intervals. GAD-7 = Generalized Anxiety Disorder; EMM = estimated marginal means. The x-axis reflects sessions (primarily weekly) of treatment, with 0 = baseline, 1 = session 1, etc. 1MFU = is the 1 month follow up assessment which ranged from 2 to 5 weeks after end of treatment.

Based on GAD-7 scale, there was a significant linear reduction in anxiety over time. When modeling time as a continuous predictor, bootstrapped estimates showed anxiety decreased by approximately 0.50 points per session (*B* = −0.50, *SE* = 0.08, *p* < 0.01). The fixed effect of time alone explained 12.22% of the variance in GAD-7 scores; However, the full model which included individual baseline differences, explained 78.38% of the variance. Categorical analyses demonstrated decreases in anxiety scores at multiple time points relative to baseline (*M* = 10.50, *SE* = 1.14). Statistically significant differences based on bootstrapped CIs in EMM scores for anxiety emerged consistently at session 8 through 10 and at session 12 (Figure 7). Specifically, GAD-7 scores at mid-point (session 6) did not indicate significant decline (*M* = 6.92, *SE* = 0.96, *Hedges g* = 1.08 [0.16, 2.16]) based on bootstrapped CIs; however, end of treatment (*M* = 3.11, *SE* = 0.90, *Hedges g* = 2.22 [0.80, 4.04]), and one-month follow-up (*M* = 4.72, *SE* = 2.30, *Hedges g* = 1.74 [0.54, 3.22]) indicated significant declines from baseline.

### 3.8. Adverse Events

There were no serious adverse events that occurred in this study. An independent Data Safety and Monitoring Board reviewed all adverse events at annual meetings.

## 4. Discussion

This Stage IA pilot study aimed to test the preliminary feasibility, acceptability, and promise of a modified, integrated, trauma-focused treatment, Helping Opioid Use Disorder and PTSD with Exposure (HOPE) among *N* = 6 individuals with co-occurring OUD/PTSD and stabilized on medications for OUD (Onken et al., 2014). The primary research questions assessed whether participants receiving HOPE showed significant within-subject reductions in PTSD symptoms, opioid use and craving, retention on medications for OUD, and client satisfaction throughout treatment. Secondary outcomes assessed if participants receiving HOPE showed reductions in anxiety and depression symptoms throughout treatment. Results showed that participants in HOPE showed significant reductions in PTSD symptoms as well as high client satisfaction. There were no significant changes in medication for OUD due to overall high compliance throughout treatment. One participant used opioids, and another participant reported misuse of their medication for OUD. Despite this use, both participants successfully stabilized to their prescribed medication for OUD doses by end of treatment and follow-up. Contrary to the hypotheses, opioid use and all other substance use did not significantly change throughout treatment. However, craving significantly decreased by 1-month follow-up, but not by end of treatment. To our knowledge, this is the first study to examine an integrated, trauma-focused treatment among individuals with OUD/PTSD. The implication of these pilot findings are further discussed below.

### 4.1. PTSD Outcomes

Findings from this pilot study were promising reducing PTSD symptoms across both self-reported and clinician-rated PTSD symptom measures. Results continue to suggest that exposure-based therapies, which include imaginal exposure and in vivo exposures, are successful in reducing PTSD symptoms. However, novel, in this study, was the adaptation from the traditional Prolonged Exposure therapy and COPE protocols in that sessions did not require imaginal exposure and in vivo exposures at each session following their introduction into the treatment. Rather, during the development of HOPE, stakeholders and patients expressed a desire for titration of imaginal and in vivo exposures. Findings herein show that such titration still yields significant reductions in PTSD symptoms at end of treatment and follow up. Indeed, a recent study testing Prolonged Exposure therapy with financial incentives in people with OUD/PTSD showed an average decrease of 18.3 points on the CAPS-5 and 24.8 points on the PCL-5 by end of treatment (Peck et al., 2025). In the current study, the average decrease in CAPS-5 symptoms was 15 points from baseline to end of treatment and 18.4 points from baseline to follow up. Further the decrease in the PCL-5 was 24.48 from baseline to end of treatment and 26.52 points from baseline to follow up. As such, at face value, this study shows similar clinically significant reductions in PTSD symptoms as Prolonged Exposure with financial incentives in Peck et al. (2025) (Marx et al., 2021).

An interesting trend noticed throughout the analyses was the reduction of PTSD symptoms during the latter half of the HOPE therapy. Namely, reductions in PTSD symptoms on the CAPS-5 were only significant at end of treatment and at follow up. In addition, weekly assessments on the PCL-5 also showed the significance of session 8 in the reduction of PTSD symptoms. Clinically, mid-treatment and session 8 correspond with the completion of 1–3 imaginal exposures for participants in the study. For one participant in particular, imaginal exposure was difficult to complete and was not achieved until session 6 (as opposed to session 4). Understandable experiences of avoidance and distress in completing the imaginal exposure likely delayed PTSD symptom reductions and again highlight the importance of imaginal exposure in alleviation of PTSD symptoms and trauma-related distress. Furthermore, session 8 also corresponded with one participant using opioids and one misusing their medication for OUD. Results also showed that session 4 had the lowest client satisfaction. Session 4 is when the first imaginal exposure is completed. The low client satisfaction ratings demonstrate that completing the first imaginal exposure may yield dissatisfaction with that therapy session, but that the satisfaction rebounds in subsequent sessions.

### 4.2. Opioid and Substance Use Outcomes

When it came to opioid use and substance use outcomes, this study found that patients continued using medications for OUD at end of treatment and follow-up and neither opioid use nor other substance use increased throughout the study. Akin to other trials among OUD/PTSD clients (e.g., Peck et al., 2025), overall endorsement of opioid use throughout the study was very low with only two participants endorsing use throughout the study. This is likely due to all participants being stabilized on medications for OUD which significantly reduces opioid use and craving. However, it is important to note that, for both participants who reported using opioids or misusing medications for OUD, there were errors or delays in the receipt of their medication for OUD (i.e., vivitrol and buprenorphine). The correspondence of these opioid lapses with medication for OUD noncompliance underscores the critical role medications for OUD play in reducing opioid craving and urges to use (Wakeman et al., 2020). Additionally, the use of vivitrol, methadone, and buprenorphine among study participants in HOPE suggests that HOPE is well tolerated among different types of extant medications for OUD.

Although it is promising that there were no significant increases in substance use among participants in this pilot study, the lack of significant reductions in opioid use and all other substance use was contrary to study hypotheses and can be interpreted in several ways. On the one hand, the absence of significant increases in substance use suggests that the trauma-focused therapy of HOPE was well tolerated among individuals with OUD/PTSD and on medications for OUD without showing an increase in substance use. However, these findings also suggest that additional content on reducing substance use could be included in future iterations of the HOPE protocol to facilitate additional substance use reductions. Alternatively, it may be fruitful to consider, in future works, changes to standard measures for assessing substance use to better assess the frequency and quantity of polysubstance use across time periods. In this study, the focus on average using days (i.e., the average days a participant used a particular substance) may have subverted the detection of changes in the amount of substance use per using day, a measurement challenge in polysubstance use (e.g., Bunting et al., 2024; Hindocha et al., 2018). These questions will be important to answer in future research among a larger participant sample in a randomized clinical trial.

Nevertheless, it was promising to see the statistically significant reduction in the desire for opioids by follow-up. The delayed reductions in craving may be due to reductions in PTSD symptoms first being necessary for subsequent change in substance use outcomes to occur (e.g., Hien et al., 2018). As such, decreases in PTSD symptoms in HOPE may have impacted decreases in the desire for opioids by follow-up, but this plausible mechanism must be tested empirically. The delayed decrease in desire for opioids may also have implications for considerations regarding treatment length (i.e., ~16 sessions) or treatment delivery (phases) that first focuses on PTSD reductions while maintaining low substance use followed by focusing more intently on substance use reductions in latter sessions may be beneficial to patients with OUD/PTSD. Importantly, some of this study’s findings were contradictory. Results showed significant decreases in the desire for opioids on the desire for drugs questionnaire (in both continuous and categorical analyses), but these findings were not evident on the opioid craving scale in both model types. One possible reason for this discrepancy may be simply terminology—many patients did not report in the moment craving of opioids on the craving scale but reported desiring the effects of opioids on the desire for drug questionnaire completed at baseline, mid-treatment, and end-of-treatment. As discussed in the HOPE therapy, it is possible that patients were aware that cravings for opioids will ebb and flow, but throughout treatment the overall desire to use reduced given patients’ commitment to their respective recoveries. In all, decreases in the desire for opioid use are taken as preliminary and require future replication.

### 4.3. Client Satisfaction

Additional outcomes that are noteworthy to mention include the high retention in the HOPE therapy and overall client satisfaction. Present research shows that dropout rates from most integrated, trauma-focused treatments remain moderately high at values hovering near ~40–60% (e.g., Belleau et al., 2017; Szafranski et al., 2019). Although this study’s findings need to be replicated in a larger sample, the overall dropout rate was significantly lower than these studies at 16.7% and retention was 83.3%. One possible reason for higher retention rates in this study was the flexibility with which HOPE was delivered—a vocalized desire by treatment providers and patients during treatment development (Saraiya et al., 2024). HOPE maintained flexibility in the administration of imaginal and in vivo exposures as well as session content. This flexibility prioritizes attending to presenting patient concerns at each session (e.g., self-harming behaviors, suicidality, lapses on medications for OUD or opioid use, unstable housing, medical problems) over strict adherence to manualized protocols that may sacrifice treatment alliance and retention. In this way, as with other treatments for high intensity, high risk populations, clinical needs always take priority. HOPE was developed with these safety-oriented principles in mind. The goal is that by prioritizing patient needs and empowering providers flexibility in how content is delivered throughout the therapy (rather than strictly at one specific session), alliance, trust, and retention can be increased. Indeed, this pilot study showed higher retention outcomes than financial incentives combined with Prolonged Exposure therapy (Peck et al., 2025) which may warrant greater consideration of flexibility within fidelity (Chu & Kendall, 2009; Galovski et al., 2024).

### 4.4. Secondary Outcomes

Additional promising outcomes from this pilot study were the reductions in symptoms of anxiety and depression and the increase in client satisfaction. Akin to other trauma-focused therapies, participants who received HOPE showed reductions in both anxiety and depression symptoms by end of treatment and follow up (Peck et al., 2025). Average end of treatment scores suggest that participants in this study were moderately anxious and depressed at the beginning of treatment and self-reported scores in the minimal to mild depression and anxiety range by end of treatment. These results align with extant research further demonstrating the breadth of both integrated and non-integrated trauma-focused therapies on not only efficaciously reducing PTSD symptoms, but also depression and anxiety symptoms (e.g., Aderka et al., 2013; Back et al., 2019).

### 4.5. Limitations and Future Directions

There are notable limitations to factor into the study’s findings. First, as a pilot study, the sample size was very small, and results are preliminary and require replication in a larger randomized clinical trial, which is ongoing (e.g., ClinicalTrials.govID: NCT06641115). In addition, there was no control group in this sample, and thus solely within-group effects could be observed. As such, we are not able to conclude from this study’s design whether observed reductions are due to the intervention or other third-variable factors (e.g., time, natural environment). A randomized controlled trial will allow assessment of if the combination of HOPE with medications for OUD provides greater reductions than on medication for OUD only. Further, the first author was a study therapist for the study, which may have increased bias in the delivery of HOPE to each client. Some bias was reduced by study assessors being blind to the study intervention. Fidelity checklists were completed after each session, but fidelity monitoring is not reported herein since the aim of this Stage IA pilot study, in line with the NIH Stage Model of Behavioral Therapies Development (Onken et al., 2014), was to test the initial HOPE manual and include iterative modifications for a future randomized controlled clinical trial. Nevertheless, the lack of blinding of both the study therapist and assessors—a difficult feat in all psychotherapy research trials—remains a limitation. Furthermore, it is critical to highlight the lack of diversity in the current sample. No participants identified as Black/African American or Asian/Asian American, despite members of the study team identifying with these racial/ethnic identities. The lack of racial/ethnic representativeness in this sample mirrors work showing that these two communities are some of the least likely to seek substance use disorder, specifically opioid use, care (Banks et al., 2023). Thus, future work is needed on specifically increasing the engagement of Black and Asian communities into trauma-focused OUD care, which may also call for modification of the HOPE therapy itself. Modifications could include the discussion of racial trauma and examining how opioid use may be related to racism, racial trauma, and microaggressions experienced. 

Another limitation to be noted is that there are significant limitations in the field in measuring the frequency of polysubstance use across samples using a combination of alcohol, drug, and tobacco products. Efforts to increase concordant measurement across substances will significantly enhance the ability to detect substance use outcomes. Similarly, ensuring the validity of trauma-focused assessments among individuals with OUD will be important for future work to consider given that, observationally, some trauma assessments were difficult for participants to complete since they seemed to lack awareness of their trauma/PTSD symptoms. Indeed, PTSD symptoms were lower in this sample than in other co-occurring OUD/PTSD samples (e.g., Mills et al., 2007). It is unclear why PTSD symptoms were lower in this sample, but in addition to possible lack of ‘awareness’ among participants, being stabilized on medications for OUD may have reduced PTSD severity. It will be important to examine this in a larger sample.

Despite these limitations, these preliminary results from a Stage IA trial are promising for advancing treatment of co-occurring OUD/PTSD among individuals taking medications for OUD. In this pilot study, HOPE showed positive outcomes among multiple domains of psychological functioning without significant changes in substance use or opioid use. In addition, client satisfaction was extremely high, as evidenced by the high retention rate in the study. Altogether, this study’s findings suggest that in a trauma-focused, integrated treatment where the number of exposures was reduced, sessions lasted 60-min, content was delivered flexibly, and overall treatment varied in length from 10–12 sessions, the therapy was deemed acceptable, feasible, and shows early preliminary efficacy among individuals with OUD/PTSD and stabilized on medications for OUD. Given the early state of this therapy, the next ongoing steps will be to test the efficacy of HOPE more rigorously in a randomized clinical trial.

## Figures and Tables

**Table 1:** Participant demographics (*N* = 6).

Variable	*N* (%) or *M* (SD)
Age	44.7 (12.9)
Sex	
Woman	4 (66.7%)
Man	2 (33.3%)
Racial Identity	
White	4 (66.7%)
Multiracial (Native American/White)	1 (16.7%)
Pacific Islander	1 (16.7%)
Native American/Alaska Native	0 (0%)
Black/African American	0 (0%)
Asian	0 (0%)
Hispanic/Latino (Central American origin)	1 (16.7%)
Have children (yes/no)	4 (66.7%)
Monthly income	$1650 ($1764)
Employment (Part-time or Full-time)	5 (83.3%)
Insurance	
Medicaid	5 (83.3%)
Private	1 (16.7%)
Education	
Some high school	1 (16.7%)
Completed high school/GED	4 (66.7%)
Some college or Associates	1 (16.7%)
Incarceration History	4 (66.7%)
Religion	
Christianity/Catholicism	3 (50.0%)
Islam	1 (16.7%)
Atheist	2 (33.3%)
Relationship status	
Single/never married	3 (50.0%)
Living with Partner	1 (16.7%)
Divorced	1 (16.7%)
Children (yes)	4 (66.7%)
Number of children	1.4 (1.1)
Primary caregiver of children (yes)	1 (16.7%)

**Table 2:** Clinical characteristics (*N* = 6).

Variable	*N* (%) or *M* (SD)
Medication for OUD	
Methadone	1 (16.7%)
Buprenorphine/Suboxone	4 (66.7%)
Naltrexone/Vivitrol	1 (16.7%)
Age of first opioid use	19.7 (7.2)
Age of opioid use disorder onset	21.3 (6.7)
Ever overdosed (yes/no)	1 (16.7%)
Current diagnoses	
Current OUD	6 (100.0%)
Major depressive episode	2 (33.3%)
Alcohol use disorder	3 (50.0%)
Sedative use disorder	3 (50.0%)
Cannabis use disorder	2 (33.3%)
Stimulant use disorder	3 (50.0%)
Nicotine User	5 (83.3%)
Chronic pain	2 (33.3%)
PHQ-9 at baseline	12.0 (5.1)
GAD-7 at baseline	10.5 (4.3)
*PTSD Severity*	
CAPS-5 at baseline	32.0 (3.1)
PCL-5 at baseline	42.3 (7.5)
*Trauma Characteristics*	
Lifetime trauma exposure	8.7 (1.5)
Index trauma	
Sexual assault	3 (50.0%)
Physical assault	2 (33.3%)
Sudden violent death	1 (16.7%)
Natural disaster, fire/explosion, transportation accident, other serious accident, or exposure to toxic substance	0 (0%)
Assault with a weapon	0 (0%)
Other unwanted or uncomfortable sexual experience	0 (0%)
Combat, exposure to a war-zone, or captivity	0 (0%)
Life-threatening illness or injury	0 (0%)
Severe human suffering	0 (0%)
Sudden violent death	0 (0%)
Sudden accidental death	0 (0%)
Serious injury, harm, or death to someone else	0 (0%)
Childhood Trauma Questionnaire	
Emotional abuse subscale	13.3 (2.8)
Physical abuse subscale	11.8 (2.9)
Sexual abuse subscale	13.2 (3.5)
Emotional neglect subscale	14.7 (1.3)
Physical neglect subscale	12.8 (5.9)

## Data Availability

Data is available upon request to the corresponding author.

## References

[B1-behavsci-15-00874] Aderka I. M., Gillihan S. J., McLean C. P., Foa E. B. (2013). The relationship between posttraumatic and depressive symptoms during prolonged exposure with and without cognitive restructuring for the treatment of posttraumatic stress disorder. Journal of Consulting and Clinical Psychology.

[B2-behavsci-15-00874] American Psychiatric Association (2013). Diagnostic and statistical manual of mental disorders.

[B3-behavsci-15-00874] Attkisson C. C., Zwick R. (1982). The client satisfaction questionnaire. Psychometric properties and correlations with service utilization and psychotherapy outcome. Evaluation, Programming, and Planning.

[B4-behavsci-15-00874] Back S. E., Foa E. B., Killeen T., Mills K., Teesson M., Dansky Cotton B., Carroll K. M., Brady K. T. (2014). Concurrent treatment of PTSD and substance use disorders using prolonged exposure (COPE): Therapist Manual.

[B5-behavsci-15-00874] Back S. E., Killeen T., Badour C. L., Flanagan J. C., Allan N. P., Ana E. S., Lozano B., Korte K. J., Foa E. B., Brady K. T. (2019). Concurrent treatment of substance use disorders and PTSD using prolonged exposure: A randomized clinical trial in military veterans. Addictive Behaviors.

[B6-behavsci-15-00874] Ballinger G. A. (2004). Using generalized estimating equations for longitudinal data analysis. Organizational Research Methods.

[B8-behavsci-15-00874] Banks D. E., Brown K., Saraiya T. C. (2023). “Culturally responsive” substance use treatment: Contemporary definitions and approaches for minoritized racial/ethnic groups. Current Addiction Reports.

[B7-behavsci-15-00874] Bates D., Mächler M., Bolker B., Walker S. (2015). Fitting linear mixed-effects models using lme4. Journal of Statistical Software.

[B9-behavsci-15-00874] Becker W. C., Ganoczy D., Fiellin D. A., Bohnert A. S. (2015). Buprenorphine/Naloxone dose and pain intensity among individuals initiating treatment for opioid use disorder. Journal of Substance Use Treatment.

[B10-behavsci-15-00874] Belleau E. L., Chin E. G., Wanklyn S. G., Zambrano-Vazquez L., Schumacher J. A., Coffey S. F. (2017). Pre-treatment predictors of dropout from prolonged exposure therapy in patients with chronic posttraumatic stress disorder and comorbid substance use disorders. Behavior Research Therapy.

[B11-behavsci-15-00874] Bernstein D. P., Fink L. (1998). Childhood Trauma Questionnaire: A retrospective self-report manual.

[B12-behavsci-15-00874] Biondi B. E., Zheng X., Frank C. A., Petrakis I., Springer S. A. (2020). A literature review examining primary outcomes of medication treatment studies for opioid use disorder: What outcome should be used to measure opioid treatment success?. American Journal of Addiction.

[B13-behavsci-15-00874] Blevins C. A., Weathers F. W., Davis M. T., Witte T. K., Domino J. L. (2015). The posttraumatic stress disorder checklist for DSM-5 (PCL-5): Development and initial psychometric evaluation. Journal of Traumatic Stress.

[B14-behavsci-15-00874] Bunting A. M., Shearer R., Linden-Carmichael A. N., Williams A. R., Comer S. D., Cerdá M., Lorvick J. (2024). Are you thinking what I’m thinking? Defining what we mean by “polysubstance use.”. American Journal of Drug and Alcohol Abuse.

[B15-behavsci-15-00874] Center for Disease Control and Prevention (2018). Annual surveillance report of drug-related risks and outcomes—United States.

[B16-behavsci-15-00874] Chu B. C., Kendall P. C. (2009). Therapist responsiveness to child engagement: Flexibility within manual-based CBT for anxious youth. Journal of Clinical Psychology.

[B17-behavsci-15-00874] Cohen J. A., Mannarino A. P. (2015). Trauma-focused cognitive behavior therapy for traumatized children and families. Child and Adolescent Psychiatric Clinic of North America.

[B18-behavsci-15-00874] Cottler L. B., Compton W. M., Mager D., Spitznagel E. L., Janca A. (1992). Posttraumatic stress disorder among substance users from the general population. American Journal of Psychiatry.

[B19-behavsci-15-00874] Danovitch I. (2016). Post-traumatic stress disorder and opioid use disorder: A narrative review of conceptual models. Journal of Addictive Diseases.

[B20-behavsci-15-00874] Ecker A. H., Hundt N. (2018). Posttraumatic stress disorder in opioid agonist therapy: A review. Psychological Trauma: Theory, Research, Practice and Policy.

[B21-behavsci-15-00874] First M. B., Williams J. B. W., Karg R. S., Spitzer R. L. (2015). Structured clinical interview for DSM-5—Research version (SCID-5 for DSM-5, research version; SCID-5-RV).

[B22-behavsci-15-00874] Foa E. B., Hembree E. A., Rothbaum B. O., Rauch S. A. M. (2019). Prolonged exposure therapy for PTSD: Emotional processing of traumatic experiences—Therapist guide.

[B23-behavsci-15-00874] Franken I. H., Hendriksa V. M., van den Brink W. (2002). Initial validation of two opiate craving questionnaires the obsessive compulsive drug use scale and the desires for drug questionnaire. Addictive Behaviors.

[B24-behavsci-15-00874] Galovski T. E., Nixon R. D. V., Kehle-Forbes S. (2024). Walking the line between fidelity and flexibility: A conceptual review of personalized approaches to manualized treatments for posttraumatic stress disorder. Journal of Traumatic Stress.

[B25-behavsci-15-00874] Gardner W., Mulvey E. P., Shaw E. C. (1995). Regression analyses of counts and rates: Poisson, overdispersed Poisson, and negative binomial models. Psychological Bulletin.

[B26-behavsci-15-00874] Haller M., Chassin L. (2014). Risk pathways among traumatic stress, posttraumatic stress disorder symptoms, and alcohol and drug problems: A test of four hypotheses. Psychology of Addictive Behaviors.

[B27-behavsci-15-00874] Hassan A. N., Le Foll B., Imtiaz S., Rehm J. (2017). The effect of post-traumatic stress disorder on the risk of developing prescription opioid use disorder: Results from the National Epidemiologic Survey on Alcohol and Related Conditions III. Drug and Alcohol Dependence.

[B29-behavsci-15-00874] Hien D. A., Smith K. Z., Owens M., Lopez-Castro T., Ruglass L. M., Papini S. (2018). Lagged effects of substance use on PTSD severity in a randomized controlled trial with modified prolonged exposure and relapse prevention. Journal of Consulting and Clinical Psychology.

[B30-behavsci-15-00874] Hindocha C., Norberg M. M., Tomko R. L. (2018). Solving the problem of cannabis quantification. The Lancet Psychiatry.

[B28-behavsci-15-00874] Højsgaard S., Lauritzen S. (2024). On some algorithms for estimation in Gaussian graphical models. Biometrika.

[B31-behavsci-15-00874] Hser Y. I., Saxon A. J., Huang D., Hasson A., Thomas C., Hillhouse M., Jacobs P., Teruya C., McLaughlin P., Wiest K., Cohen A., Ling W. (2014). Treatment retention among patients randomized to buprenorphine/naloxone compared to methadone in a multi-site trial. Addiction.

[B32-behavsci-15-00874] Kadden R., Carroll K., Donovan D., Cooney N., Monti P., Abrams D., Litt M., Hester R. (1992). Cognitive-behavioral coping skills therapy manual: A clinical research guide for therapists treating individuals with alcohol abuse and dependence.

[B33-behavsci-15-00874] Khoury L., Tang Y. L., Bradley B., Cubells J. F., Ressler K. J. (2010). Substance use, childhood traumatic experience, and Posttraumatic Stress Disorder in an urban civilian population. Depressin and Anxiety.

[B34-behavsci-15-00874] Kroenke K., Spitzer R. L. (2002). The PHQ-9: A new depression and diagnostic severity measure. Psychiatric Annals.

[B35-behavsci-15-00874] Lenth R. (2025). Emmeans: Estimated marginal means, aka least-squares means. *(R package version 1.11.1-00001)*.

[B36-behavsci-15-00874] Marx B. P., Lee D. J., Norman S. B., Bovin M. J., Sloan D. M., Weathers F. W., Keane T. M., Schnurr P. P. (2021). Reliable and clinically significant change in the clinician-administered PTSD Scale for DSM-5 and PTSD Checklist for DSM-5 among male veterans. Psychological Assessment.

[B37-behavsci-15-00874] McHugh R. K., Fitzmaurice G. M., Carroll K. M., Griffin M. L., Hill K. P., Wasan A. D., Weiss R. D. (2014). Assessing craving and its relationship to subsequent prescription opioid use among treatment-seeking prescription opioid dependent patients. Drug and Alcohol Dependence.

[B38-behavsci-15-00874] McHugh R. K., Hilton B. T., Chase A. M., Griffin M. L., Weiss R. D. (2021). Do people with opioid use disorder and posttraumatic stress disorder benefit from adding individual opioid drug counseling to buprenorphine?. Drug and Alcohol Dependence.

[B39-behavsci-15-00874] Meier A., Lambert-Harris C., McGovern M. P., Xie H., An M., McLeman B. (2014). Co-occurring prescription opioid use problems and posttraumatic stress disorder symptom severity. American Journal of Drug and Alcohol Abuse.

[B40-behavsci-15-00874] Meshberg-Cohen S., Black A. C., DeViva J. C., Petrakis I. L., Rosen M. I. (2019). Trauma treatment for veterans in buprenorphine maintenance treatment for opioid use disorder. Addictive Behaviors.

[B41-behavsci-15-00874] Mills K. L., Teesson M., Ross J., Darke S. (2007). The impact of post-traumatic stress disorder on treatment outcomes for heroin dependence. Addiction.

[B42-behavsci-15-00874] Mills K. L., Teesson M., Ross J., Peters L. (2006). Trauma, PTSD, and substance use disorders: Findings from the Australian national survey of mental health and well-Being. American Journal of Psychiatry.

[B43-behavsci-15-00874] Nelson E. C., Heath A. C., Lynskey M. T., Bucholz K. K., Madden P. A. F., Statham D. J., Martin N. G. (2006). Childhood sexual abuse and risks for licit and illicit drug-related outcomes: A twin study. Psychological Medicine.

[B44-behavsci-15-00874] Nguyen T. D., Attkisson C. C., Stegner B. L. (1983). Assessment of patient satisfaction: Development and refinement of a service evaluation questionnaire. Evaluation, Programming, and Planning.

[B45-behavsci-15-00874] Onken L. S., Carroll K. M., Shoham V., Cuthbert B. N., Riddle M. (2014). Reenvisioning clinical science: Unifying the discipline to improve the public health. Clinical Psychological Science.

[B46-behavsci-15-00874] Parida S., De Aquino J. P., Sofuoglu M. (2019). High potency synthetic opioids: Curbing the third wave of the opioid crisis. Neuroscience and Biobehavioral Reviews.

[B47-behavsci-15-00874] Peck K. R., Giannini J., Badger G. J., Cole R., Sigmon S. C. (2025). A novel prolonged exposure therapy protocol for improving therapy session attendance and PTSD symptoms among adults receiving buprenorphine or methadone treatment. Drug and Alcohol Dependence.

[B48-behavsci-15-00874] Peck K. R., Moxley-Kelly N., Badger G. J., Sigmon S. C. (2021). Posttraumatic stress disorder in individuals seeking treatment for opioid use disorder in vermont. Preventitive Medicine.

[B49-behavsci-15-00874] Peck K. R., Schumacher J. A., Stasiewicz P. R., Coffey S. F. (2018). Adults with comorbid posttraumatic stress disorder, alcohol use disorder, and opioid use disorder: The effectiveness of modified Prolonged Exposure. Journal of Traumatic Stress.

[B50-behavsci-15-00874] Peirce J. M., Brooner R. K., King V. L., Kidorf M. S. (2016). Effect of traumatic event reexposure and PTSD on substance use disorder treatment response. Drug and Alcohol Dependence.

[B51-behavsci-15-00874] Sadeh N., Miglin R., Bounoua N., Beckford E., Estrada S., Baskin-Sommers A. (2021). Profiles of lifetime substance use are differentiated by substance of choice, affective motivations for use, and childhood maltreatment. Addictive Behaviors.

[B52-behavsci-15-00874] Santo T., Campbell G., Gisev N., Tran L. T., Colledge S., Di Tanna G. L., Degenhardt L. (2021). Prevalence of childhood maltreatment among people with opioid use disorder: A systematic review and meta-analysis. Drug and Alcohol Dependence.

[B53-behavsci-15-00874] Saraiya T. C., Helpinstill S., Gray D., Hien D. A., Brady K. T., Hood C. O., Back S. E. (2024). The lived experiences and treatment needs of women with opioid use disorder and PTSD symptoms: A mixed-methods study of women and providers. Journal of Substance Use and Addiction Treatment.

[B54-behavsci-15-00874] Schacht R. L., Brooner R. K., King V. L., Kidorf M. S., Peirce J. M. (2017). Incentivizing attendance to prolonged exposure for PTSD with opioid use disorder patients: A randomized controlled trial. Journal of Consulting and Clinical Psychology.

[B55-behavsci-15-00874] Schiff M., Levit S., Cohen-Moreno R. (2010). Childhood sexual abuse, post-traumatic stress disorder, and use of heroin among female clients in Israeli methadone maintenance treatment programs (MMTPS). Social Work & Health Care.

[B56-behavsci-15-00874] Schiff M., Nacasch N., Levit S., Katz N., Foa E. B. (2015). Prolonged Exposure for treating PTSD among female methadone patients who were survivors of sexual abuse in Israel. Social Work & Health Care.

[B57-behavsci-15-00874] Sloan D. M., Marx B. P. (2019). Written exposure therapy for PTSD: A brief treatment approach for mental health professionals.

[B58-behavsci-15-00874] Smith K. Z., Smith P. H., Cercone S. A., McKee S. A., Homish G. G. (2016). Past year non-medical opioid use and abuse and PTSD diagnosis: Interactions with sex and associations with symptom clusters. Addictive Behaviors.

[B59-behavsci-15-00874] Sobell L. C., Sobell M. B., A. P. Association (2000). Alcohol timeline followback (TFLB). Handbook of psychiatric measures.

[B60-behavsci-15-00874] Spitzer R. L., Kroenke K., Williams J. B., Löwe B. (2006). A brief measure for assessing generalized anxiety disorder: The GAD-7. Archives of Internal Medicine.

[B61-behavsci-15-00874] Szafranski D. D., Gros D. F., Acierno R., Brady K. T., Killeen T. K., Back S. E. (2019). Heterogeneity of treatment dropout: PTSD, depression, and alcohol use disorder reductions in PTSD and AUD/SUD treatment noncompleters. Clinical Psychology & Psychotherapy.

[B62-behavsci-15-00874] Volkow N., Benveniste H., McLellan A. T. (2018). Use and misuse of opioids in chronic pain. Annual Review of Medicine.

[B63-behavsci-15-00874] Wakeman S. E., Larochelle M. R., Ameli O., Chaisson C. E., McPheeters J. T., Crown W. H., Azocar F., Sanghavi D. M. (2020). Comparative effectiveness of different treatment pathways for opioid use disorder. JAMA Network Open.

[B66-behavsci-15-00874] Weathers F. W., Blake D. D., Schnurr P. P., Kaloupek D. G., Marx B. P., Keane T. M. (2013a). The life events checklist for DSM-5 (LEC-5).

[B67-behavsci-15-00874] Weathers F. W., Bovin M. J., Lee D. J., Sloan D. M., Schnurr P. P., Kaloupek D. G., Keane T. M., Marx B. P. (2018). The Clinician-Administered PTSD scale for DSM-5 (CAPS-5): Development and initial psychometric evaluation in military veterans. Psychological Assessment.

[B68-behavsci-15-00874] Weathers F. W., Litz B. T., Keane T. M., Palmieri P. A., Marx B. P., Schnurr P. P. (2013b). The PTSD Checklist for DSM-5 (PCL-5).

[B64-behavsci-15-00874] Wickham H., Averick M., Bryan J., Chang W., McGowan L. D., François R., Grolemund G., Hayes A., Henry L., Hester J., Kuhn M., Pedersen T. L., Miller E., Bache S. M., Müller K., Ooms J., Robinson D., Seidel D. P., Spinu V., Yutani H. (2019). Welcome to the tidyverse. Journal of Open Source Software.

[B65-behavsci-15-00874] Wickham H., Miller E., Smith D. (2023). Haven: Import and export ‘SPSS’, ‘Stata’ and ‘SAS’ files. *(R package version 2.5.3)*.

